# Balloon catheter‐assisted endoscopic resection for papillary adenoma of non‐exposed protruded type (with video)

**DOI:** 10.1002/deo2.408

**Published:** 2024-07-15

**Authors:** Weigang Gu, Justin Ryan L. Tan, Hangbin Jin, Qifeng Lou, Chuang Tang, Ka Shing Cheung, Jianfeng Yang, Xiaofeng Zhang

**Affiliations:** ^1^ Department of Gastroenterology Affiliated Hangzhou First People's Hospital Hangzhou China; ^2^ Department of Gastroenterology Chinese General Hospital and Medical Center Manila Philippines; ^3^ Department of Gastroenterology Metropolitan Medical Center Manila Philippines; ^4^ Department of Medicine The University of Hong Kong‐Shenzhen Hospital Shenzhen China; ^5^ Queen Mary Hospital Hong Kong Hong Kong

**Keywords:** ampullectomy, endoscopic resection, intraductal extension, papillary adenoma, papillectomy

## Abstract

Papillary adenomas, known precursors to papillary adenocarcinoma, warrant close monitoring due to their malignant potential. Historically, surgical resection represented the mainstay of treatment for papillary adenomas with intraductal extension. However, recent advancements in endoscopic techniques have facilitated the adoption of endoscopic papillectomy as a minimally invasive alternative in carefully selected cases. We report a case of an 82‐year‐old woman with a diagnosis of papillary adenoma exhibiting intraductal extension. This was managed with a novel endoscopic technique, balloon catheter‐assisted endoscopic resection. Due to the obscured intraductal component of the papillary mass, a balloon occlusion catheter was deployed within the common bile duct and used as traction to facilitate endoscopic visualization of the mass. Endoscopic resection via papillectomy was subsequently performed. Histopathological examination of the resected specimen revealed a villous adenoma with high‐grade dysplasia. Serial endoscopic ultrasound examinations with targeted papillary biopsies were performed to monitor for disease recurrence.

## INTRODUCTION

Papillary adenomas represent a well‐recognized precursor lesion for papillary adenocarcinoma, exhibiting a documented adenoma‐carcinoma sequence. Given their malignant potential, papillary adenomas necessitate a high index of suspicion and early intervention with curative intent.^1^, ^2^ Endoscopic papillectomy (EP) has emerged as a minimally invasive approach for achieving curative resection in select cases of papillary adenoma. However, for papillary adenomas demonstrating bile duct involvement, surgical resection remains the standard treatment.^3^ The therapeutic coverage for EP has recently been expanded to encompass papillary neoplasms with limited intraductal extension (≤20 mm).^4^ This case report details the successful management of a non‐exposed papillary adenoma with intraductal extension through the use of a balloon catheter and endoscopy polypectomy snare.

## CASE REPORT

**FIGURE 1:**
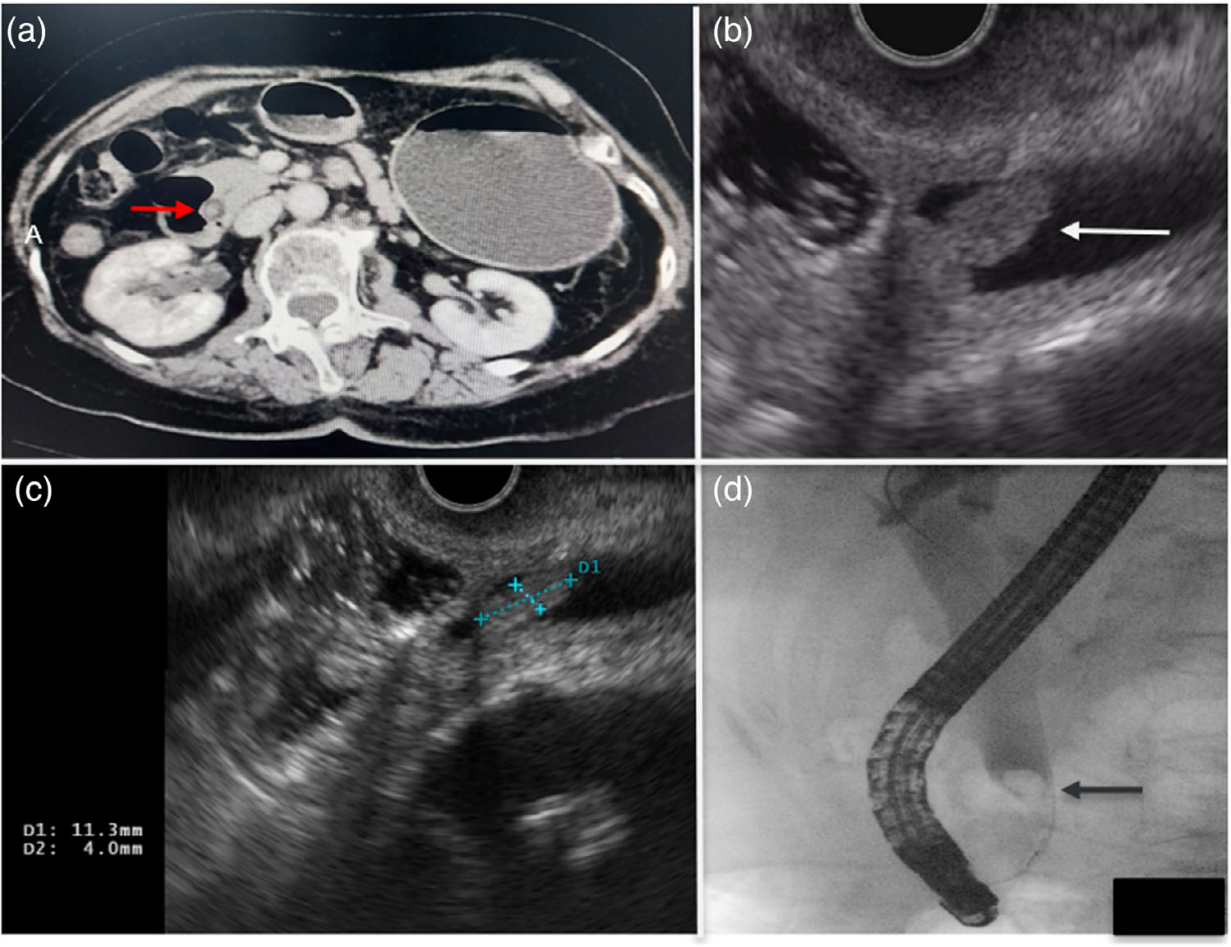
(a) Contrast‐enhanced abdominal computed tomography scan showing distal common bile duct polypoid lesion (red arrow). (b) Endoscopic ultrasound revealed a papillary mass with intraductal extension (white arrow). (c) Intraductal extension of the papillary mass measured 1.13 × 0.4 cm on endoscopic ultrasound. (d) Cholangiogram showed a filling defect within the papilla of Vater, extending proximally into the intraductal portion of the common bile duct.

**FIGURE 2:**
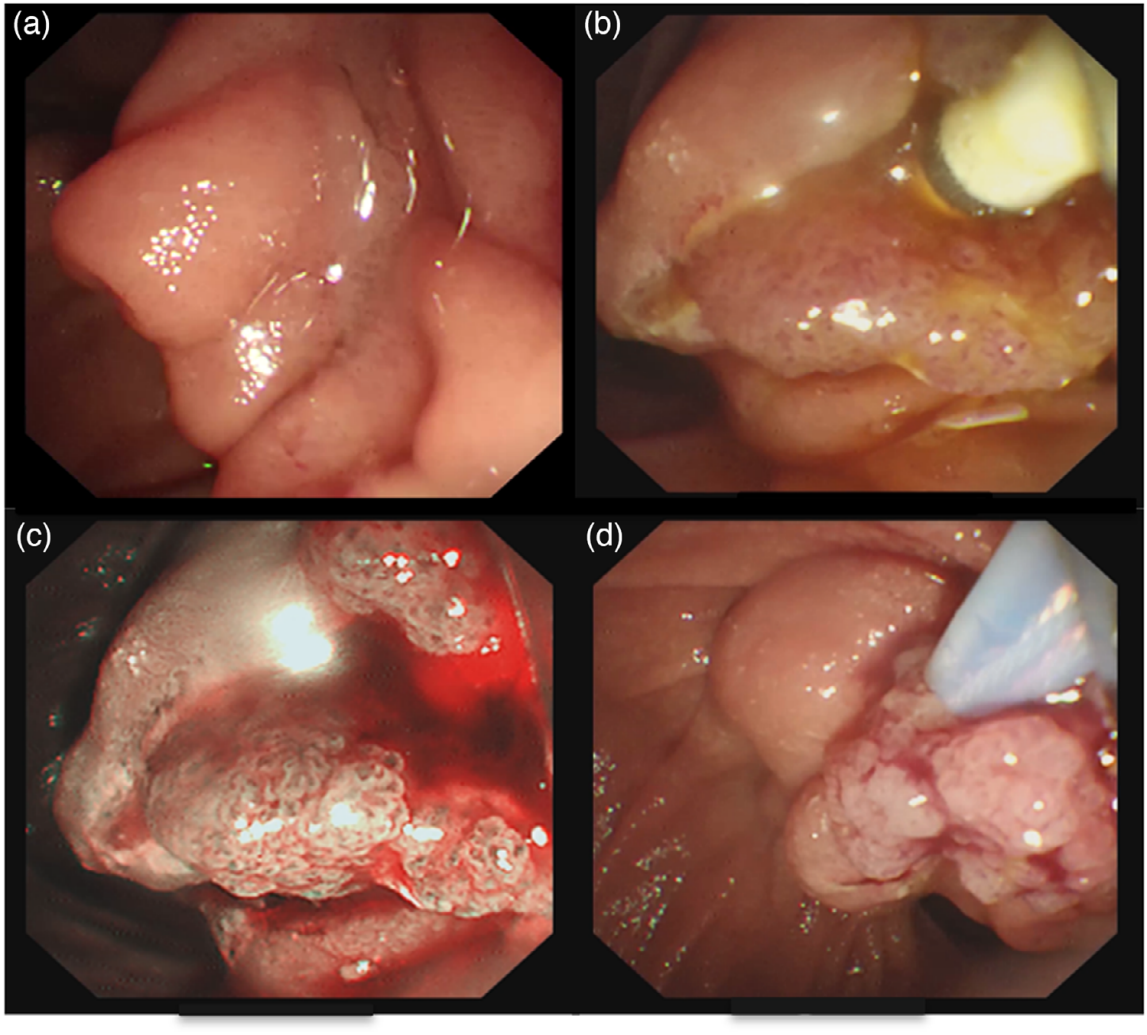
(a) Duodenal papilla with small papillary projections and uneven surface texture. (b) Balloon catheter‐assisted traction of papillary mass after sphincterotomy. (c) Narrow band imaging revealed an adenoma‐like pattern of the papillary mass. (d) Papillectomy using an electrocautery‐assisted polypectomy snare.

**FIGURE 3:**
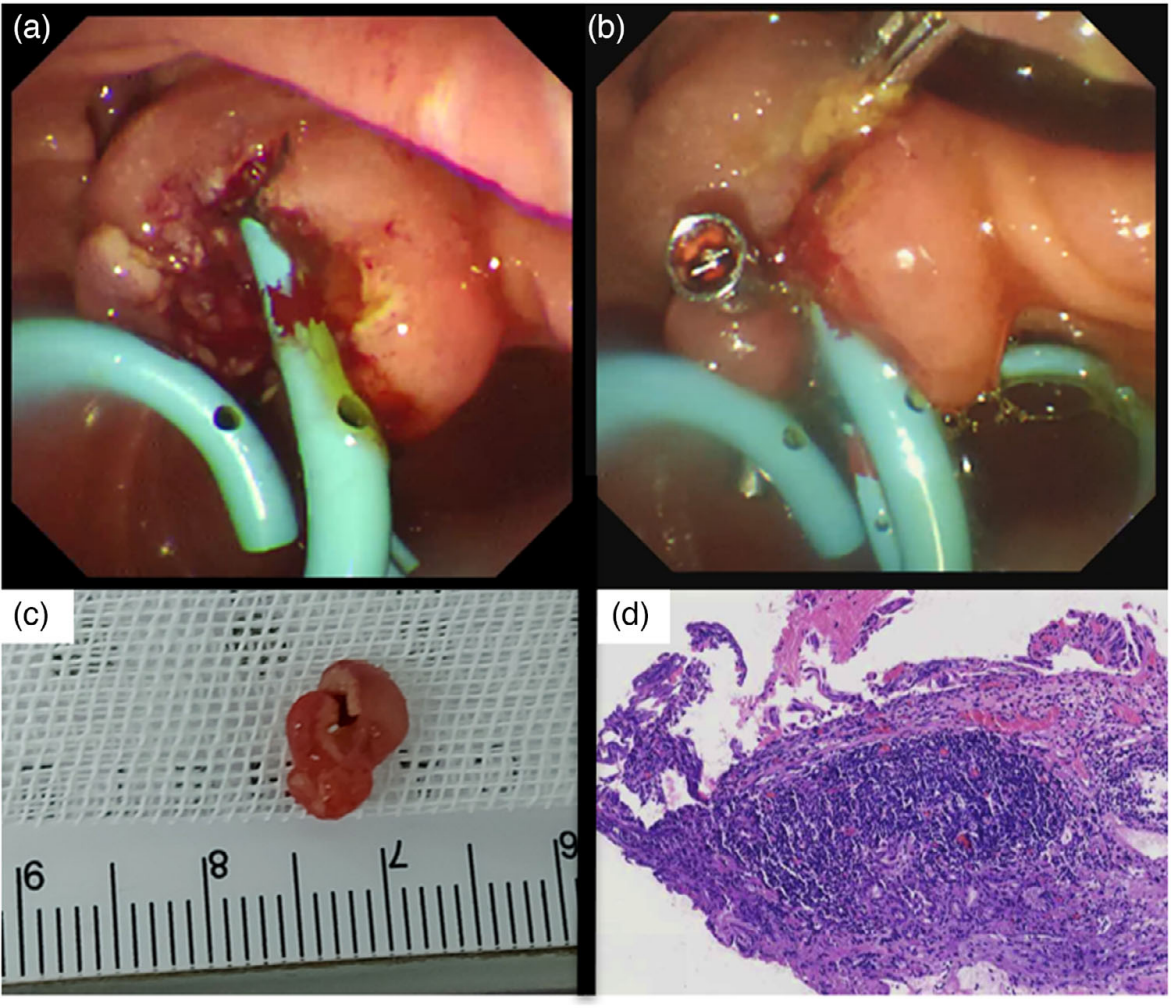
(a) Common bile duct and pancreatic duct plastic stents were inserted post‐papillectomy. (b) Hemoclips deployed for closure of the post‐resection defect. (c) The largest fragment of a papillary mass specimen following piecemeal resection. (d) Villous adenoma with high‐grade intraepithelial neoplasia (Specimen under high power field).

**FIGURE 4:**
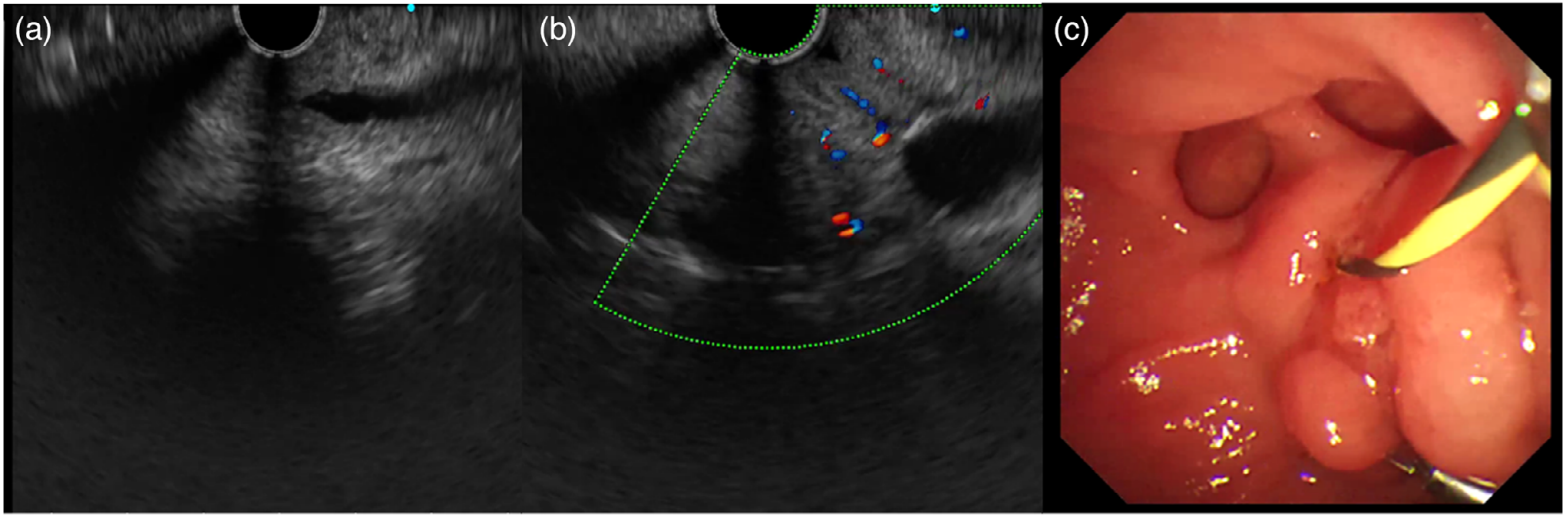
(a) Common bile duct and papilla 2 months post‐papillectomy showed no residual lesion on endoscopic ultrasound. (b) Examination of the common bile duct during endoscopic ultrasound (higher magnification). (c) Papilla 2 months post‐papillectomy.

An 82‐year‐old female was admitted due to a 3‐week history of dull abdominal pain, decreased appetite, and irregular bowel movement. The abdomen was soft, and non‐tender on physical examination. Blood exam showed elevated total bilirubin 36.5 µmol/L (N.V. = 3.4–20.5), direct bilirubin 23.8 µmol/L (0–6.84), and gamma‐glutamyl transferase 185 U/L (N.V. = 7–45). Contrast‐enhanced computed tomography (CT) scan revealed a 0.5cm isodense nodule at the distal common bile duct (CBD) with upstream dilation of the CBD and intrahepatic ducts (Figure 1a). Endoscopic ultrasound (EUS) further showed a dilated CBD and an isoechoic polypoid mass at the papilla (Figure 1b) with intraductal extension to the CBD measuring 1.13 × 0.4 cm (Figure 1c). A multidisciplinary team (MDT) discussion was done to discuss surgical management options considering the well‐documented malignant potential of papillary masses.^1^, ^2^ However, given the patient's advanced age and limited performance status, endoscopic retrograde cholangiopancreatography (ERCP) with papillary tissue acquisition for histopathologic analysis was prioritized. ERCP was performed, demonstrating a dilated CBD on cholangiography. In addition, a filling defect was identified within the papilla of Vater, extending proximally into the intraductal portion of the CBD (Figure 1d). Examination of the duodenal papilla revealed the presence of small papillary projections with an uneven surface texture, but the intraductal extension of the papillary mass was not seen on duodenoscopy (Figure 2a). The limitations associated with conventional techniques in acquiring adequate histopathological specimens due to the intraductal extent of the papillary mass necessitated informed consent from the patient's relative for endoscopic resection using a novel approach. A biliary sphincterotomy was performed, followed by the use of a fusion extraction balloon (Cook Medical) to facilitate traction of the papillary mass towards the duodenum (Figure 2b). Utilizing the balloon catheter, our team successfully exposed the papillary mass to the duodenal side (Video S1). Further assessment with narrow‐band imaging demonstrated an adenoma‐like pattern on the surface of the lesion (Figure 2c). EP through piecemeal resection was performed using a combination of electrocautery‐assisted polypectomy snare (Anrei Medical; Figure 2d) and endoscopic forceps. The placement of plastic stents within the CBD and pancreatic duct was done for prophylaxis of post‐procedural CBD stricture and pancreatitis, respectively (Figure 3a). Following biliary and pancreatic duct stenting, additional tissues surrounding the papilla were resected using electrocautery‐enhanced polypectomy snare and endoscopic forceps. Multiple hemoclips were deployed to close the post‐resection defect (Figure 3b). The largest resected specimen, measuring 0.8 × 0.5 cm (Figure 3c), along with several additional tissue samples, was submitted for examination. Histopathological analysis revealed a villous adenoma with high‐grade intraepithelial neoplasia (Figure 3d). At the 2‐month follow‐up period, EUS was performed to assess for any residual disease. EUS did not reveal any evidence of residual adenoma (Figure 4a,b). Subsequently, ERCP (Figure 4c) was performed with the placement of a plastic CBD stent to prevent stricture. During the ERCP, a biopsy was also obtained from the papilla. Histopathological examination of the biopsy specimen demonstrated no evidence of the previous lesion.

## DISCUSSION

The efficacy of EP for papillary adenoma with intraductal extension remains a subject of debate. While EP offers a less invasive approach compared to surgery, its suitability for lesions with intraductal extension is contested due to concerns about incomplete resection.^3^ Recent studies suggest the potential for EP to be a safe and effective treatment option for papillary adenomas with intraductal extension and even in unexposed intraductal growing papillary masses.^5^, ^6^ Balloon‐assisted endoscopic papillectomy was previously demonstrated in two studies to achieve exposure of unexposed intraductal growing papillary adenomas.^5^, ^6^ Aiura et al. described the balloon‐assisted endoscopic papillectomy using the “2 devices in 1 channel” approach. This method involves utilizing the single duodenoscope channel for both balloon catheter manipulation and snare resection.^6^ Our team adopted a technique similar to Kim et al., utilizing a balloon catheter for initial adenoma exteriorization followed by snare resection. This approach achieved the therapeutic goal within a single‐instrument channel configuration, eliminating the need for concomitant use of two accessories within the duodenoscope channel.^5^ The selection of subsequent techniques and instrumentation remains contingent upon the characteristics of the adenoma's intraductal extension following balloon‐mediated exteriorization.^5^, ^6^ In scenarios where complete exteriorization is not achieved, a combined approach utilizing simultaneous balloon traction and snare resection may be necessary.^6^ A potential post‐procedural concern in our case included the possibility of residual papillary adenoma tissue necessitating re‐intervention. En‐bloc resection of the papillary adenoma was not achieved in this instance due to restricted endoscopic visualization and instrument maneuverability within the papilla. Piecemeal resection carries an increased risk of late recurrence due to the potential for positive margins. This necessitates a thorough histopathological evaluation of the resected fragments and a rigorous follow‐up program for the detection of recurrent disease.^7^ In the study by Choi et al., piecemeal resection emerged as a significant independent predictor of non‐curative resection. This suggests that en bloc resection serves as a crucial protective factor for achieving complete resection, while positive pathological margins did not exert a statistically significant influence in their study.^7^

The patient's initial follow‐up EUS and subsequent histologic examination performed at 2 months post‐resection did not reveal any signs suggestive of the remnant of the papillary adenoma. Our ongoing management plan entails close endoscopic surveillance through serial EUS and magnetic resonance imaging with magnetic resonance cholangiopancreatography examinations.

Post‐EP patients necessitate extended surveillance due to their propensity for recurrence. Lee et al. implemented a rigorous follow‐up protocol, patients underwent follow‐up endoscopy at designated intervals: 1 month, 3 months, 6 months, and then every 6 months or annually for a minimum of 5 years. The follow‐up assessments included laboratory evaluations, duodenoscopy with targeted biopsies, and CT scans. CT scans were also obtained at 3, 6, and 12 months post‐EP, followed by yearly intervals thereafter. In instances where residual or recurrent neoplasia was identified during subsequent duodenoscopies, endoscopic biopsies were done.^8^

In anticipation of potential stricture formation, the team elected for placement of a biliary plastic stent at the second‐month follow‐up. An expert consensus acknowledged the potential utility of stent deployment in facilitating subsequent visualization of the distal CBD in the setting of post‐EP residual tissue. However, consensus regarding the routine use of CBD stents in post‐EP has not been achieved.^9^ Subsequent follow‐up endoscopy for this patient is scheduled at 6 months following resection. Intraductal radiofrequency ablation (RFA) will be considered as a potential therapeutic intervention if a recurrent adenoma is identified. In the recent European Society of Gastrointestinal Endoscopy guideline, the use of complementary techniques such as thermal ablation by cystotome, or RFA with temporary biliary stenting, is recommended for papillary adenoma with ≤20‐mm intraductal extension.^4^ In the recent meta‐analysis by Landim et al, RFA demonstrated promise as a viable modality for the management of post‐EP residual or recurrent lesions with intraductal extension. However, robust long‐term follow‐up data and high‐quality studies are still necessary to definitively establish its efficacy and long‐term safety profile.^10^

## CONFLICT OF INTEREST STATEMENT

None.

## ETHICS STATEMENT

‐Approval of the research protocol by an Institutional Reviewer Board (N/A)

‐Informed Consent‐ The written consent of the patient has been obtained

‐Registry and the Registration No. of the study/trial (N/A)

‐Animal Studies (N/A)

## Supporting information

VIDEO S1 Balloon catheter‐assisted papillectomy for papillary adenoma with intraductal extension.
